# The influence of label co-occurrence and semantic similarity on children’s inductive generalization

**DOI:** 10.3389/fpsyg.2015.01146

**Published:** 2015-08-17

**Authors:** Bryan J. Matlen, Anna V. Fisher, Karrie E. Godwin

**Affiliations:** ^1^Engineering, and Mathematics Program, Technology, Science, WestEd STEM Program, Redwood City, CA, USA; ^2^Department of Psychology, Carnegie Mellon University, Pittsburgh, PA, USA

**Keywords:** cognitive development, inductive generalization, semantic development, label co-occurrence

## Abstract

Semantically-similar labels that co-occur in child-directed speech (e.g., bunny-rabbit) are more likely to promote inductive generalization in preschoolers than non-co-occurring labels (e.g., lamb-sheep). However, it remains unclear whether this effect stems from co-occurrence or other factors, and how co-occurrence contributes to generalization. To address these issues, preschoolers were exposed to a stream of semantically-similar labels that don’t co-occur in natural language, but were arranged to co-occur in the experimental setting. In Experiment 1, children exposed to the co-occurring stream were more likely to make category-consistent inferences than children in two control conditions. Experiment 2 replicated this effect and provided evidence that co-occurrence training influenced generalization only when the trained labels were categorically-similar. These findings suggest that both co-occurrence information and semantic representations contribute to preschool-age children’s inductive generalization. The findings are discussed in relation to the developmental accounts of inductive generalization.

## Introduction

A distinctive feature of human cognition is the ability to make inferences that are based on knowledge of taxonomic hierarchies. For example, if told that a robin has omat genes, which would be more likely to have omat genes, a penguin or a squirrel? While no definitive answer can be reached given the available data, one might infer that the penguin is more likely to have this property, as it belongs to the category that includes robins but not squirrels (i.e., *birds*). Such category-based induction is thought to provide a powerful tool by which to navigate the world (e.g., Gelman and Markman, 1986; Osherson et al., 1990; Murphy and Ross, 2010).

Prior research has documented adults’ consistent use of category information in inductive generalization, but a key question concerns the developmental course of this ability (Badger and Shapiro, 2012). Many studies investigating this question have concluded that, similar to adults, young children’s inductive generalizations are based on identifying a common category that includes the presented items (e.g., Gelman and Coley, 1990; Gelman and Medin, 1993; Welder and Graham, 2001; Jaswal, 2004; Booth et al., 2005; Jaswal and Markman, 2007). For example, in their seminal study, Gelman and Markman, 1986; Experiment 2) provided preschool-age children the opportunity to generalize a property from two test items (e.g., a “*squirrel*” and a “*bunny*”) to a target object (e.g., a “*rabbit*”). The stimuli were selected such that visual information alone was insufficient to make the inference and category information was conveyed by synonymous^1^ labels (e.g., *bunny–rabbit*). The results indicated that children made category-based inferences at above chance level.

Traditionally, these findings have been interpreted as evidence that children’s generalizations are *category-based*: Putatively, the only reason children would consistently generalize properties from a “*bunny*” to a “*rabbit*” is because these labels refer to similar kinds. However, it has been recently proposed that some synonymous labels used in prior research not only referred to objects of similar kind but also were likely to co-occur in child-directed speech (Fisher, 2010). According to the CHILDES database (MacWhinney, 2000) the following label-pairs used in Gelman and Markman’s (1986) study co-occurred in natural speech of children or their caregivers: *bunny-rabbit*, *puppy-dog*, and *kitty-cat*; whereas other label-pairs used in this study were unlikely to co-occur (e.g., *rock-stone*, *cobra-snake*, *desert-sand*). This label co-occurrence provided another source of information (in addition to semantic similarity) on which to base an inference.

To examine whether children’s generalizations vary as a function of label co-occurrence, Fisher et al. (2011) presented a group of 4-year-old children with a property induction task with both co-occurring synonyms (e.g., *bunny-rabbit*) and non-co-occurring synonyms (e.g., *alligator-crocodile*); label type was manipulated within-participants. Importantly, following the property induction task children’s knowledge of all labels used in the study was tested in a task similar to the Peabody Picture Vocabulary Test (Dunn and Dunn, 1997). Despite apparent familiarity with all of the labels used in the study (i.e., children’s accuracy in the vocabulary knowledge test was 99% for both types of labels) 4-year-old children were more likely to provide category-consistent responses in the co-occurring labels condition than in the non-co-occurring labels condition (74%, above chance, and 51%, no different from chance, respectively). In contrast, adults were nearly at ceiling in giving category-consistent responses with both co-occurring and non-co-occurring labels. Analyses of individual patterns of responses revealed that only a small minority of 4-year-old children (35% of the sample) consistently provided category-consistent responses with non-co-occurring labels, whereas these same children were significantly more likely to provide category-consistent responses with co-occurring labels (75% of the sample).

A follow-up study replicated these findings, and ruled out the possibility that children’s knowledge of offspring-parent relations underlies children’s superior induction performance with co-occurring synonyms (Godwin et al., 2013). For example, in this study 100% of 4-year-old children were able to correctly identify a “*sheep*” as “*lamb’s mother*” in a kinship knowledge task. However, the same children were unlikely to make category-consistent inferences in a property induction task (i.e., the rate of category-consistent inferences for the *lamb-sheep* trial was only 50%). Similar to the Fisher et al. (2011) study, the same children succeeded in making inferences with co-occurring labels, with the rate of category-consistent responses ranging from 75 to 90% for co-occurring label-pairs.

Taken together, these studies suggest that category-consistent responding on generalization tasks is limited to small subset of synonymous labels. However, two issues remain unresolved. First, it is unclear whether differences in performance with co-occurring and non-co-occurring labels observed in prior studies stemmed from label co-occurrence or from other factors. Second, if co-occurrence indeed contributes to children’s generalization, the mechanism of this effect remains unclear.

In this paper, we consider two possible ways in which co-occurrence information may contribute to inductive generalization. One possibility has been articulated in Fisher (2010) and Fisher et al. (2011). Specifically, co-occurrence has been shown to give rise to strong lexical associations (Brown and Berko, 1960; McKoon and Ratcliff, 1992); therefore, it is possible that children’s generalization with co-occurring labels was the result of associative lexical priming rather than category-based reasoning. Under this interpretation, when children extend a property of a “*bunny*” to a “*rabbit*,” it is not because they reason that bunnies and rabbits are the same kind of animal; rather, the label “*bunny*” may prime the label “*rabbit*” during the course of the task. A strong version of this proposal suggests that co-occurrence information alone, in the absence of high semantic similarity, may be sufficient to promote generalization via associative lexical priming. For brevity, we will refer to this possibility as the Co-occurrence-Only Hypothesis.

Another possibility is that co-occurrence statistics interact with semantic similarity. In natural language, label co-occurrence and semantic similarity are not independent: Words with similar meaning tend to occur in similar linguistic contexts. Landauer and Dumais (1997) proposed that semantic representations can be extracted based solely on the co-occurrence statistics in speech input through the process referred to as Latent Semantic Analysis. Landauer and Dumais used long-range co-occurrence statistics (i.e., co-occurrences within a window of up to 70 words) to successfully simulate acquisition of new vocabulary in school-age children. Older children and adults may be able to take advantage of long-range co-occurrences to refine the existing representations or build new ones. For example, if an article discussing rare orchids mentions that *miltonia* is native to Brazil and Argentina, one’s representation of *orchids* may become elaborated to include *miltonia*. However, young children may only be able to take advantage of short-range co-occurrence statistics due to limitations in working and short-term memory. Under this interpretation, label co-occurrence may influence inductive generalization by increasing semantic similarity; therefore, co-occurrence information alone (i.e., when overlap in semantic features is low) may be insufficient to influence children’s generalization. We will refer to this possibility as the Interaction Hypothesis.

The present study was designed to address both issues discussed above. Specifically, in the present study, we directly tested whether co-occurrence training can influence preschoolers’ inductive generalization with semantically similar labels that failed to elicit reliable category-consistent responding in prior research (Fisher, 2010; Fisher et al., 2011; Godwin et al., 2013). Another goal was to examine how co-occurrence may contribute to generalization. Toward these goals, we used an experimental approach often employed in the statistical learning literature (e.g.,Saffran et al., 1996, 1998; Aslin et al., 1998). In statistical learning studies, participants are typically exposed to an auditory speech stream consisting of a string of repeating nonsense syllables that comprise words of an artificial language. Within the speech stream, some of the syllables have low transitional probabilities, whereas other syllables have high transitional probabilities. The speech stream is often designed such that the only cues to the word boundaries are the transitional probabilities of each syllable. Following exposure to the speech stream, the participants’ task is to discriminate between “words” and “part-words” of the artificial language.

Transitional probability is one way of capturing the co-occurrence relation of units in a language (Aslin et al., 1998). In the present study we adapted the statistical learning paradigm by replacing nonsense syllables with non-co-occurring semantically similar labels. We exposed preschool-age children to a speech stream that consisted of four semantically similar label-pairs that are highly familiar to young children but unlikely to co-occur in natural language (e.g., *dolphin-whale*, *sofa-couch*, *mountain-hill*, *glove-mitten*). The transitional probability within each label-pair was 100% and the transitional probability between the pairs was 33% (Co-occurrence Training). In Experiment 1, a second speech stream consisting of the same label-pairs was created, but the labels were arranged such that the semantically similar labels did not co-occur despite being presented at equal frequency to the co-occurring stream (Frequency Training). After being exposed to one of the two speech streams, children were presented with an inductive generalization task identical to that in Fisher et al. (2011) and Godwin et al. (2013). If label co-occurrence influences children’s inductive inferences, children who were exposed to the co-occurring stream should be more likely to choose category-consistent test items than children who were exposed to the frequency stream, as well as a group of children who were not exposed to either speech stream (No Training Baseline). We also predicted that, because the Frequency Training consisted of semantically similar labels that had low transitional probability, children in the Frequency Training condition would perform no different than chance level. These predictions were tested in Experiment 1. Experiment 2 replicated the findings of Experiment 1 and tested the source of co-occurrence effects on generalization (i.e., Co-occurrence-Only Hypothesis vs. the Interaction Hypothesis) by presenting children with a training speech stream in which co-occurrence was induced for semantically dissimilar items.

## Experiment 1

### Materials and Methods

#### Participants

Participants were 62 four-year-old children (*M* = 4.55 years, *SD* = 0.33 years, 32 females, 30 males) recruited from preschools in a large metropolitan area. The Institutional Review Board at Carnegie Mellon University approved the study, and parents/guardians of all children provided informed written consent before participation. One child was excluded from analysis because of a developmental delay reported by the teachers. Participants were randomly assigned to the Co-occurrence Training condition (*N* = 19), the Frequency Training condition (*N* = 21), or the No Training Baseline condition (*N* = 21).

#### Materials

Language materials consisted of four label triads, with each triad comprised of a Target item, a Category-choice item, and a Lure (see Table 1). Two of the triads referred to natural kind items and the other two referred to artifacts. Target and Category-choice items represented a subset of language materials used in a prior study (Fisher et al., 2011). This subset of items was chosen because prior research confirmed that: (1) older children and adults generalize properties from Targets to Category-choice test items for the chosen synonyms (Fisher et al., 2011); (2) 4-year-old children are highly familiar with the Target and Category-choice items (i.e., in prior work, 4-year-old children exhibited an average accuracy of 96.5% across Target and Category-choice items when presented with a picture identification task similar to the Peabody Picture Vocabulary Test; Dunn and Dunn, 1997); and (3) these labels never co-occurred in the five CHILDES databases we examined, indicating that they are unlikely to co-occur in child-directed speech.

**TABLE 1:** **Complete list of linguistic stimuli (note that each set was presented twice during the induction phase, paired with a different blank predicate on each presentation)**.

**Trial no**	**Target items**	**Related test items**	**Lures**	**Blank predicates**
1	Sofa	Couch	Chair	Matlen
2	Mountain	Hill	Forest	Troxel
3	Dolphin	Whale	Seal	Creighan
4	Glove	Mitten	Sweater	Koski
5	Mountain	Hill	Forest	Erwin
6	Sofa	Couch	Chair	Lignin
7	Glove	Mitten	Sweater	Higa
8	Dolphin	Whale	Seal	Omat

To-be-generalized properties consisted of two-syllable blank predicates. Lures were selected on the basis that they were of the same ontological kind as the Target and Category-choice items, but of a different basic level category (similar to the lures used in Gelman and Markman, 1986). To confirm that the Target items were more semantically similar to the Category-choice items than to the Lures, we conducted a separate calibration study with a group of 18 adults. Adults were asked to rate the semantic similarity of label pairs on a 7 point scale, with “7” indicating that the labels could be used interchangeably, and “1” indicating that the labels had no overlap in meaning. There were 144 filler label pairs and 12 experimental label pairs. Filler pairs consisted of a range of labels, some expected to be high in semantic similarity (e.g., alligator—crocodile), items expected to be medium in semantic similarity (e.g., alligator—octopus), and items expected to be low in semantic similarity (e.g., alligator—telephone)^2^. Experimental label pairs consisted of all Target and Category-choice items used in the present study (e.g., *dolphin-whale*; four label pairs) and all Target and Lure items (e.g., *dolphin-seal* and *whale-seal*; eight label pairs); experimental label-pairs were randomly interspersed among the filler label-pairs. Participants’ scores were averaged across all Target and Category-choice items and across all Target and Lure items. Results of the calibration confirmed that Target and Category-choice items used in the present study were rated as more semantically similar to each other (*M* = 5.86) than to the Lures (*M* = 4.22), paired-sample *t* (17) = 8.5, *p* < 0.001.

**FIGURE 1:**
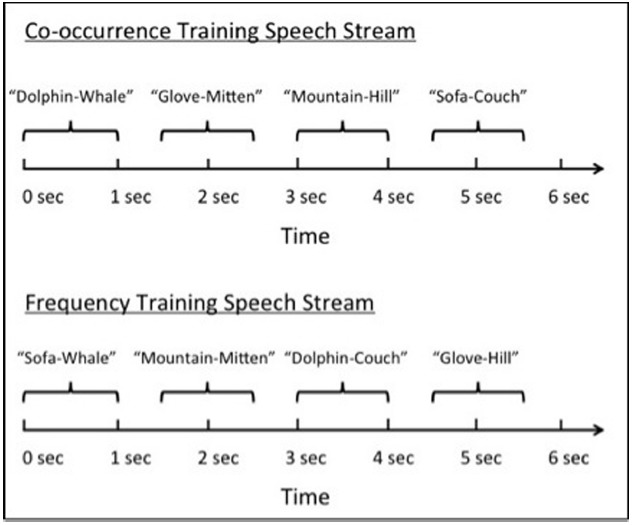
**Schematic depiction of the Co-occurrence Training speech stream (top) and Frequency Training speech stream (bottom)**.

To create the Co-occurring speech stream in which Target and Category-choice items co-occurred, a female native English speaker was recorded pronouncing each of the four semantically similar label-pairs individually^3^. Each recording was then edited to last approximately one second in duration and these recordings were used to create a speech stream in which each semantically similar label-pair occurred a total of 75 times. A short pause that lasted approximately 500 ms was included in between each label-pair, and label-pairs were arranged such that they had an equal probability of occurring next to any other label-pair (33%; see Figure 1). The speech stream lasted for a total of 7.5 min.

To create the Frequency speech stream in which Target and Category-choice items did not co-occur, all possible combinations of Target and Category-choice items from the Co-occurring stream were used, but were rearranged such that Targets were never paired with their Category-choice item: Instead, Targets were paired with the Category-choice items of the other semantically similar pairs. For example, the word “*dolphin*” was paired with the words “*hill*,” “*mitten*,” and “*couch*,” whereas the word “*whale*” was paired with the words “*mountain*,” “*glove*,” and “*sofa*.” The same female native English speaker that produced the Co-occurring stream was recorded pronouncing each label-pair individually (there were 12 label-pairs in total) and each recording was then edited to last approximately one second in duration. A speech stream was then created in which each label-pair occurred a total of 25 times: therefore, each individual label occurred a total of 75 times (the same label frequency as in the Co-occurrence Training condition). A short pause that lasted approximately 500 ms was included in between each label-pair, and the label-pairs were arranged such that any given label never occurred directly adjacent to its semantically similar counterpart (see Figure 1). As in the Co-occurring stream, the Frequency speech stream lasted a total of 7.5 min.

Visual stimuli for the induction task consisted of three sets of doors, with each set including three identical doors. Participants were told that the objects were hiding behind each of the doors. The objects were never depicted. This procedure was utilized to ensure that semantic similarity was the only source of information on which to base inferences (Fisher et al., 2011; Godwin et al., 2013).

#### Procedure

Children were tested individually in a quiet room at their school. Children were randomly assigned to the Co-occurrence Training, Frequency Training, or the No Training Baseline conditions. The experiment consisted of two parts: a listening phase and an induction phase. Only children in the Co-occurrence Training and the Frequency Training conditions participated in the listening phase, whereas all children participated in the induction phase. In the listening phase, the experimenter told children that they would be coloring pictures and that afterward, that they would play a game. Coloring was chosen as an activity as it does not require verbal working memory resources and therefore did not interfere with their potential to process the speech stream. At the same time, this activity kept children entertained for the duration of the speech stream. Once children began coloring, the experimenter started playing the speech stream on a laptop computer. Children were exposed to either the Co-occurrence speech stream or the Frequency speech stream based on the child’s condition assignment. The listening phase lasted for a total of 7.5 min, which was chosen because pilot work suggested that children could remain on task for this duration of time.

In the induction phase, the experimenter told children that they were going to play a game where they would be told about different objects hiding behind doors, and that they would be asked a question about the objects. On each trial, the Target item was always hidden behind the topmost door, and the location of the Category-choice item and Lure (to the bottom left or to the bottom right of the target) was randomized across trials. The experimenter first introduced the target item (e.g., *There is a dolphin hiding behind this door*) and then introduced the other test items in random order (e.g., *There is a seal hiding behind this door*. *There is a whale hiding behind this door*). Children were then told about the property of the target item (e.g., *The dolphin has omat inside*) and were asked to generalize this property to one of the test items (e.g., *Which do you think has omat inside like this dolphin, the whale or the seal?*)^4^. The presentation order of Category-choice items and Lures was counterbalanced across trials. Participants were allowed to respond either by verbally labeling one of the test items or by pointing to one of the test doors. In total, children completed eight trials, two trials for each of the four semantically similar label-pairs. Trials were presented in one of two pseudorandom orders (either trials 1–8 in Table 1, or the reverse order).

### Results and Discussion

**FIGURE 2:**
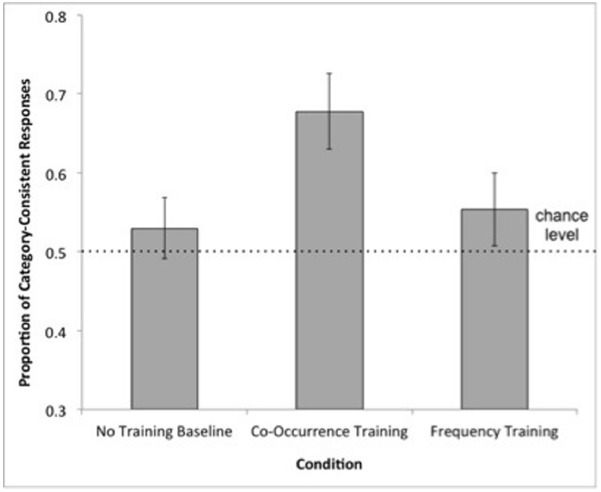
**Mean proportion of category-consistent responses by condition in Experiment 1.** Error bars represent standard errors of the means. The dotted line indicates chance performance.

To compare children’s performance against chance level, the proportion of choices of the Category-choice items was calculated for each participant and averaged across participants within each condition. In the Frequency Training (*M* = 0.55, *SD* = 0.21) and No Training Baseline (*M* = 0.53, *SD* = 0.18) conditions, the proportion of Category-choice responses did not exceed chance level (0.50), one-sample *t*s < 1.17, *p*s > 0.25. However, in the Co-occurrence Training condition (*M* = 0.67, *SD* = 0.21), children’s rate of selecting Category-choice items was significantly greater than chance, *t*(18) = 3.70, *p* < 0.005 (see Figure 2).

We compared performance between conditions using logit mixed effects regression using the lme4 package in R (Bates et al., 2013). This analysis has been used in prior developmental research (e.g., Ping and Goldin-Meadow, 2010) and is recommended over ANOVA as it can account for the nesting of individual trials within subjects, as well as handle binary outcomes (i.e., either synonym = 1 or lure = 0) (Jaeger, 2008). A varying-intercept model predicted the likelihood of category responses using condition as a fixed effect and a random effect of subject (log-likelihood = –326.19, BIC = 677.14). This model was significantly different from an unconditional model that included only the random effect of subject (*χ*^2^ = 6.45, *p* = 0.04; log-likelihood = –329.42, BIC = 671.21). The varying-intercept model was also not statistically different from a model that included condition as a fixed effect and a random intercept and triad slope for each subject (*χ*^2^ = 8.59, *p* = 0.48; log-likelihood = –321.89, BIC = 724.26) indicating that the inclusion of triad in the model does not improve the model fit. Using the Co-occurrence Training condition as a baseline, the varying-intercept model showed a significant effect of the No Training Baseline condition (β = –0.65, SE = 0.27, *p* = 0.01) and the Frequency Training condition (β = –0.55, SE = 0.27, *p* = 0.04) indicating that children were less likely to choose synonym responses in these conditions relative to the Co-occurrence Training condition (see Table 2 for a summary of fixed effects results). This effect was of medium size for the comparison with the Frequency Training condition, Cohen’s *d* = 0.57, and the No Training Baseline condition, Cohen’s *d* = 0.72.

**TABLE 2:** **Summary of the fixed effects in the mixed logit model predicting performance on the induction task in Experiment 1 (*N* = 61, log-likelihood = –326.2, BIC = 677.1). Co-occurrence Training condition served as the baseline in the model**.

	**β**	**SE**	**Wald *Z***	***p***
Intercept	0.77	0.20	3.86	0.001*
No training baseline condition	–0.65	0.27	–2.41	<0.05*
Frequency training condition	–0.54	0.27	–2.04	<0.05*

* significant at alpha < 0.05.

To test whether children in the No Training Baseline condition performed significantly different than children in the Frequency Training condition, we reran the model using the No-Training Baseline condition as the baseline. We found no effect of the Frequency Training condition (β = 0.10, SE = 0.25, *p* > 0.69), suggesting that children were not more likely to choose synonym responses in the Frequency Training condition relative to the No Training Baseline condition.

Due to the limited number of items, a proper item analysis was not possible. Nevertheless, there were no differences between performance on natural kind and artifact items (paired-sample *t* = 0.46, *p* = 0.65), which is consistent with prior findings (e.g., Fisher et al., 2011).

To summarize, children’s performance on the property induction task did not exceed chance level unless semantic similarity information was combined with co-occurrence information (Co-occurrence Training). Moreover, training condition significantly predicted children’s induction performance: Children in the Co-occurrence Training condition were more likely to choose the Category-choice response options than children in the Frequency Training condition and the No Training Baseline condition. The findings of this Experiment provide direct evidence that label co-occurrence affects inductive generalization in preschool-age children.

One outstanding question concerns whether co-occurrence information is sufficient for altering children’s generalization or whether co-occurrence statistics of the input interact with semantic similarity. We address this question in Experiment 2, in which we exposed children to a speech stream in which non-semantically similar labels co-occurred. Specifically, we took the Lures and one of the Synonymous labels from each of the triads in Experiment 1 and paired them together so that they co-occurred in a speech stream (e.g., *seal-dolphin*). After children listened to the speech stream, they participated in the inductive generalization task in which the Lure from Experiment 1 (e.g., *seal*) was described as having the novel property, and children were asked to generalize this property to one of the synonymous labels (e.g., *dolphin* or *whale*), only one of which was trained in the co-occurrence speech stream (e.g., *dolphin*). This design allowed us to control for the effect of semantic similarity between the lure and two synonymous labels, therefore isolating the effect of co-occurrence on inductive generalization. If children’s performance is affected by the co-occurrence of Target and Lure items, this would provide evidence in support of the Co-occurrence-Only hypothesis. Specifically, this finding would suggest that co-occurrence effects observed in prior inductive generalization studies were likely due to lexical priming of the category choice item by the target item. In contrast, if co-occurrence of Target and Lure items is not sufficient to affect children’s induction performance, this would provide evidence in support of the Interaction hypothesis. Such a finding would suggest that effects observed in prior inductive generalization studies stemmed from co-occurrence increasing semantic similarity of co-occurring synonyms. In addition to distinguishing between these possibilities, Experiment 2 also sought to replicate the main finding of Experiment 1 (i.e., the effect of co-occurrence training with semantically similar labels on children’s induction).

## Experiment 2

### Materials and Methods

#### Participants

Participants were 54 four-year-old children (*M* = 4.55 years, *SD* = 0.35 years, 24 females, 30 males) recruited from preschools in a large metropolitan area. The Institutional Review Board at Carnegie Mellon University approved the study, and parents/guardians of all children provided informed written consent before participation. Two children were excluded from analysis because of experimenter error. Participants were randomly assigned to the Synonym Co-occurrence Training condition (*N* = 18), the Non-Synonym Co-Occurrence Training condition (*N* = 16), or the No Training Baseline condition (*N* = 18).

#### Materials and Procedure

There were three between-subject conditions in Experiment 2: the Non-Synonym Co-occurrence Training condition, the Synonym Co-occurrence training condition, and the No Training Baseline condition. The latter two conditions were included to determine if the results of Experiment 1 would replicate with regards to the effect of co-occurrence training of synonymous labels on children’s inductive generalization. For these conditions (i.e., Synonym Co-Occurrence Training and No Training Baseline) the materials and procedure were identical to those in Experiment 1. For the Non-Synonym Co-occurrence Training condition, a speech stream was created that consisted of the Lures used in Experiment 1 and one of the labels from each synonym-pair (e.g., *seal–whale*, *forest–hill*, *chair-couch*, and *sweater-mitten*). The same female speaker from Experiment 1 recorded the label pairs. The label-pairs were sequenced within a speech stream such that trained label-pairs exhibited 100% co-occurrence probability to each other and 33% co-occurrence probability to any other pair. Each label occurred a total of 75 times (i.e., with the same frequency as in Experiment 1). The speech stream lasted a total of 7.5 min in duration (i.e., the same duration as the Synonym Co-Occurrence speech stream).

Only children in the Non-Synonym Co-Occurrence Training and the Synonym Co-Occurrence Training conditions participated in the listening phase. All children participated in the induction task. For the Synonym Co-Occurrence Training and No Training Baseline conditions, the induction task was identical to Experiment 1. For the Non-Synonym Co-Occurrence condition, children completed a modified version of the induction task in which the Target items consisted of the Lures from Experiment 1, and the Test items consisted of the two semantically similar labels from Experiment 1. For example, children could be told that a “*Seal*” had “*Creighan*” inside and asked which of the two Test items—the “*Whale*” (i.e., Trained item) or the “*Dolphin*” (i.e., Untrained item)—also had “*Creighan*” inside. To ensure that children did not exhibit a bias to choose either of the Trained or Untrained items prior to training, a separate group of 16 four-year-old children (*M* = 4.33, *SD* = 0.23) completed the Non-Synonym induction task without listening to the Non-Synonym Co-Occurrence speech stream. The results indicated that children did not choose the to-be-trained test items at levels greater than chance (*M* = 0.51, *SD* = 0.13, *p* > 0.81).

### Results and Discussion

In order to compare children’s performance against chance level, the proportion of choices of the Trained Test items was calculated for each participant and averaged across participants within each condition. In the Synonym Co-Occurrence Training and No Training Baseline Conditions, the Trained Test items were synonymous labels (e.g., *dolphin-whale*). In the Non-Synonym Co-Occurrence Training condition, the Trained Test items were non-synonymous labels (e.g., *seal-whale*).

**FIGURE 3:**
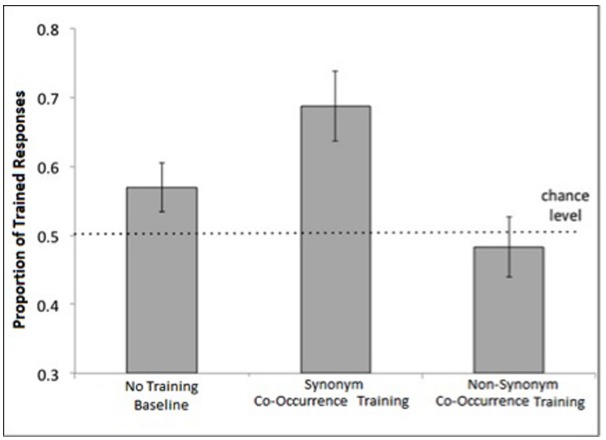
**Mean proportion of trained responses by condition in Experiment 2.** Error bars represent standard errors of the means. The dotted line indicates chance performance.

The proportion of Trained item choices in the Non-Synonym Co-Occurrence Training condition of Experiment 2 did not exceed chance [*M* = 0.49, *SD* = 0.17; *t*(15) = < 1, *ns*], suggesting that co-occurrence alone is not sufficient to induce category-consistent generalization. As in Experiment 1, children’s performance in the Synonym Co-occurrence Training condition was significantly greater than chance (*M* = 0.69, *SD* = 0.22), one-sample *t*(17) = 3.69, *p* < 0.005 (see Figure 3). The proportion of Category-choice responses in the No Training Baseline condition (*M* = 0.57, *SD* = 0.15) was not significantly above chance, one-sample *t*(17) = 1.97, *p* = 0.07.

To compare performance between conditions within Experiment 2, we predicted the likelihood of trained responses using a fixed effect for condition and a random effect for subject (log-likelihood = –276.55, BIC = 577.21). This model was significantly different from an unconditional model that included only the random effect of subject (*χ*^2^ = 9.91, *p* = 0.007; log-likelihood = –281.5, BIC = 575.06). The model was also not statistically different from a model that included the fixed effect of condition and a random intercept and triad slope for each subject (*χ*^2^ = 3.4, *p* = 0.94; log-likelihood = –274.84, BIC = 628.09) indicating that accounting for triad-type does not improve the model fit. Using the Synonyms Co-occurrence Training condition as a baseline, the varying-intercept model showed a significant effect of the No Training Baseline condition (β = –0.51, SE = 0.25, *p* = 0.04) and the Non-Synonym Co-occurrence Training condition (β = –0.82, SE = 0.26 *p* = 0.001) indicating that children were less likely to choose trained responses in these conditions relative to the Synonym Co-occurrence Training condition (see Table 3 for a summary of fixed effects results). This effect was large for the comparison with the Non-Synonym Co-Occurrence Training condition, Cohen’s *d* = 1.02, and medium for the No Training Baseline condition, Cohen’s *d* = 0.64.

**TABLE 3:** **Summary of the fixed effects in the mixed logit model predicting performance on the induction task in Experiment 2 (*N* = 52, log-likelihood = –276.5, BIC = 577.2). Co-occurrence Training condition served as the baseline in the model**.

	**β**	**SE**	**Wald *Z***	***p***
Intercept	0.79	0.19	4.28	<0.001*
No training baseline condition	–0.51	0.25	–2.03	<0.05*
Non-synonym co-occurrence training condition	–0.82	0.26	–3.18	=0.001*

* significant at alpha < 0.05.

To test whether children in the No Training Baseline condition performed significantly different than children in the Non-Synonym Co-Occurrence Training condition, we reran the model using the No-Training Baseline condition as the baseline in the model. We found no effect of the Non-Synonym Co-Occurrence Training condition (β = –0.31, SE = 0.25, *p* = 0.21). There were also no differences between performance on natural kind and artifact items (paired-sample *t* = 0.64, *p* = 0.53).

In summary, results of Experiment 2 replicate the results from Experiment 1 and extend these findings in important ways. As in Experiment 1, children in the Synonym Co-Occurrence Training condition outperformed children in the No Training Baseline condition by giving a higher proportion of Category-choice responses. However, we found no evidence that co-occurrence information alone—in the absence of substantial semantic overlap—influenced young children’s generalization behavior, as children in the Non-Synonym Co-occurrence Training condition were equally likely to choose the untrained semantically-dissimilar labels (e.g., *dolphin-seal*) as the trained semantically-dissimilar labels (e.g., *whale-seal*) in the property induction task. Importantly, it is unlikely that children made a pragmatic guess about the goal of the co-occurrence training and strategically chose the co-occurring items during the induction task: if this were the case, it is not clear why children would make this pragmatic choice in the Co-Occurring Synonyms condition (Experiments 1–2) but not in the Co-Occurring Non-Synonyms condition (Experiment 2).

## General Discussion

The results of the reported experiments replicate the prior findings that preschool-age children are unlikely to use the semantic similarity of labels to make inferences with labels that do not co-occur in the English language (i.e., No Training Baseline condition in Experiments 1–2 and Frequency Training condition in Experiment 1 of this manuscript; Fisher, 2010; Fisher et al., 2011, 2015; Godwin et al., 2013). At the same time, the present findings provide the first direct evidence that combining semantic similarity with co-occurrence information leads to an increase in category-consistent responding on a property induction task. In other words, a short training session that did not impart any new knowledge onto children but instead established an association between semantically similar labels led children to prefer category-choice items to lures on a property induction task. These findings suggest that effects previously reported in the literature (Gelman and Markman, 1986; Fisher et al., 2011; Godwin et al., 2013) can be at least partially attributed to label co-occurrence.

Importantly, results of Experiment 2 help to clarify the mechanisms of this co-occurrence effect. Specifically, brief co-occurrence training was not sufficient to promote generalization in the absence of substantial semantic overlap. The duration of co-occurrence training in the present studies was brief and it is possible that longer co-occurrence training of semantically dissimilar items may influence children’s generalization behavior. Nonetheless, the same brief co-occurrence training was sufficient to influence property induction for semantically similar items, the effect that was observed in both Experiment 1 and 2. Therefore, results of Experiment 2 undermine the Co-occurrence-Only hypothesis, which suggests that children’s success in making inductive inferences with co-occurring semantically similar labels is due to associative lexical priming (Fisher, 2010; Fisher et al., 2011).

Lexical priming occurs when a response to a word is influenced by its preceding linguistic context. These effects have been observed in a number of tasks, including lexical decision, word naming, and object naming among others (for review and discussion, see Jones and Estes, 2012). Researchers commonly distinguish between semantic and associative priming, among several other types of priming. *Associative priming* occurs when responses are influenced by local (i.e., immediate) co-occurrence of words that tend to occur together in linguistic context but not necessarily share meaning (e.g., “fire-truck”). *Pure semantic priming* refers to effects that are driven only by similarity in meaning and not by other types of relations (e.g., “dog-wolf”). Associative and semantic priming differ in their time course (i.e., semantic priming effects decay faster than associative priming effects), suggesting differences in processing of semantic and associative relations (Jones and Estes, 2012). Importantly, these two types of priming may also have different developmental trajectories. Specifically, priming based on associative relations has been shown in children as young as 24 months of age (Arias-Trejo and Plunkett, 2013). However, the evidence of purely semantic priming is mixed, with some studies reporting semantic priming effects in 24-month-olds (Arias-Trejo and Plunkett, 2013), whereas other studies report associative but not semantic priming effects at 6 years of age (McCauley et al., 1976). When semantic and associative relations are combined, evidence of priming has been observed in children as young as 21-months of age (Arias-Trejo and Plunkett, 2009), and even in adults semantic priming effects are stronger in the presence of association (Moss et al., 1995).

The above factors led Fisher (2010) and Fisher et al. (2011) to hypothesize that effects of co-occurring semantically similar labels on inductive generalization in preschoolers may stem largely from associations established by co-occurrence, rather than from the shared meaning of labels. However, results of Experiment 2 undermine this hypothesis. Instead, these results support the Interaction hypothesis, suggesting that label co-occurrence and semantic similarity interact to influence preschoolers’ inductive generalization performance.

As discussed in the introduction, semantic similarity can be extracted from linguistic context on the basis of long-range co-occurrence statistics of the input (for review, see Bullinaria and Levy, 2007). Consistent with this notion, Shapiro et al. (2012) recently demonstrated that spatiotemporal co-occurrence increases similarity of visual object representations in the medial temporal lobe in adult participants. In the present experiments, we used labels that have a high overlap in meaning but are unlikely to immediately co-occur in child-directed speech. It is likely that limitations in working and short-term memory preclude preschool-age (and younger) children from extracting semantic similarity from long-range co-occurrence statistics in the natural language (and even short-range co-occurrence statistics, i.e., the Frequency Control condition in Experiment 1). At the same time, immediate co-occurrences induced by the Synonym Co-occurrence Training condition in Experiments 1–2 may have increased semantic similarity of Target and Category-choice items, enabling children to make category-consistent responses.

It is possible that developmental improvement in working and short-term memory allow older children to build and refine semantic representations based on long-range co-occurrences in linguistic input. Initial evidence supporting this possibility has been recently reported by Vlach and Johnson (2013) who found that 16-month-old infants succeeded in mapping novel words to referents only when novel labels were presented on a *massed* but not on an *interleaved* training schedule. Trials involving novel words and referents were presented in immediate succession in the massed condition, but were separated by other items in the interleaved condition. However, at 20-months of age, infants were able to learn the novel words in both massed and interleaved conditions. It may seem implausible that 20-month-olds are able to learn new words in an interleaved learning schedule but preschoolers have trouble extracting semantic similarity of non-co-occurring labels. However, in Vlach and Johnson’s (2013) study the linguistic context was relatively simple (i.e., two novel labels and referents). Furthermore, mapping a novel label to a referent is only a first step in building semantic representations. Therefore, the contribution of memory development to children’s ability to build semantic representations based on mid- and long-range co-occurrence statistics in natural language remains to be examined in future research.

### Broader Theoretical Implications

Prior research has documented that children can make novel inferences beginning in early infancy (e.g., Mandler and McDonough, 1996; Rakison, 2007). However, it remains uncertain how children accomplish this important task and whether they accomplish it in a way that is fundamentally similar to the way in which adults and older children would accomplish an analogous task. A dominant view of children’s inductive generalization characterizes it as a category-based reasoning process, akin to that performed by adults (Gelman and Markman, 1986; Gelman and Coley, 1990; Jaswal and Markman, 2007). According to this theoretical perspective, children are “initially biased” to believe that labels refer to kinds, and that items of similar kind are likely to have similar unobservable properties (Gelman and Markman, 1986, p. 207; see also Gelman and Coley, 1990; Welder and Graham, 2001; Gelman, 2003). In the course of induction, children first determine which of the presented entities belong to the same category and then make an inference on the basis of this categorization.

An alternative perspective suggests that induction is a process of automatic generalization supported by overall featural similarity in children (e.g., Sloutsky and Fisher, 2004, 2012) and in many circumstances in adults as well (Sloman, 1993, 1996). It has been shown that in infancy categories are based on the distributions of perceptual features (e.g., Younger and Cohen, 1986; Rakison and Butterworth, 1998; French et al., 2004). In the world outside the lab, category members have many perceptual features in common; therefore, perceptual groupings built in infancy can support the formation of semantic categories as children learn through direct and indirect experience (e.g., books, TV, etc.) other features that are correlated with perceptual features (e.g., “balls roll,” “birds lay eggs,” etc.) (cf. Smith and Heise, 1992). Within this theoretical perspective, induction is not based on the consideration of which category includes the presented items, but on the summed featural similarity weighted by the salience of auditory labels in case of infants and young children (for discussion, see Sloutsky and Fisher, 2004, 2012).

In the case of preschool-age children’s inductive generalization behavior, the studies reported in this paper are partially inconsistent with both theories. Specifically, a naïve theory account cannot explain why the majority of children do not spontaneously produce category-based responses in absence of association-based training. Alternatively, a purely association-based account cannot explain why children do not respond based on trained associations, in the absence of semantic similarity. Instead, these findings are more consistent with a modified view that acknowledges both the roles of semantic and perceptual information in children’s inductive generalization.

The suggestion that both category knowledge and association/similarity contribute to children’s generalization behavior has been articulated by other developmental scientists (Waxman and Gelman, 2009). However, an ongoing debate concerns whether innate starting points (i.e., naïve theories) are necessary to explain children’s generalization behavior from birth to early childhood. The present findings are inconsistent with the possibility that category-based induction in children is an “initial bias” (Gelman and Markman, 1986). Specifically, without the co-occurrence training, the majority of 4 year-old children did not make category-consistent inferences about highly-familiar items.

At the same time, the present findings are consistent with a recently proposed theoretical account of the development of children’s inductive generalization. The Perceptual and Representational Similarity (PaRS; Fisher et al., 2015) account proposes that both knowledge and perceptual information contribute to the development of children’s inductive generalization. However, the PaRS account makes a distinction between knowledge gained through experience (i.e., *semantic knowledge*) and knowledge people may have “independent of experience” (Gelman and Markman, 1986, p. 207), such as essentialist beliefs or naïve theories (see also Gelman, 2003). Whereas the PaRS account endorses the influence of semantic knowledge in preschool children’s generalization behavior, it argues against the necessity to posit the existence of innate knowledge to account for children’s inductive inferences. In the present paper, we suggest that the co-occurrence training may have increased the representational similarity of the Target and Category-choice items in the semantic space (cf. Shapiro et al., 2012), an interpretation that is consistent with the PaRS account.

## Summary

The findings presented in this paper provide the first experimental evidence that label co-occurrence has a direct effect on inferences made by preschool-age children. Specifically, exposing 4-year-old children to a stream of words in which semantically similar labels were adjacent to each other increased category-consistent responding on a property induction task. At the same time, co-occurrence training with semantically dissimilar labels did not influence children’s generalizations, suggesting an interaction between semantic similarity and label co-occurrence. These findings provide support for the hypothesis that label co-occurrence increases semantic similarity of representations, thus promoting category-consistent responding on an inductive inference task. Future research is necessary to examine a corollary prediction of this account, namely that developmental increases in working and short-term memory enable children to take advantage of mid- and long-range co-occurrences in linguistic input to form and refine semantic representations.

### Conflict of Interest Statement

The authors declare that the research was conducted in the absence of any commercial or financial relationships that could be construed as a potential conflict of interest.

## Footnotes

1 Very few labels share 100% overlap in meaning. Following Gelman and Markman (1986), in this paper we use the term “synonymous” to refer to labels that are semantically similar rather than semantically equivalent, for the sake of brevity.

2 Ratings for filler pairs were used in a calibration of labels for a separate study (Fisher et al., 2015).

3 Statistical learning studies are often designed such that transitional probabilities are the only cues to word boundaries. Given that the focus of the current studies was on generalization rather than word segmentation, we did not eliminate co-articulation cues from the speech stream, as these cues are undoubtedly present for the label-pairs which co-occur in natural language.

4 The wording “has X inside” was used for both artifact and natural kind labels because prior work (Fisher et al., 2011) suggested that children’s inductive generalization was not affected by subtle changes in question wording (e.g., “has X inside” vs. “is made of X”).
